# Analysis of a German blood donor cohort reveals a high number of undetected SARS-CoV-2 infections and sex-specific differences in humoral immune response

**DOI:** 10.1371/journal.pone.0279195

**Published:** 2022-12-16

**Authors:** Bastian Fischer, Cornelius Knabbe, Tanja Vollmer

**Affiliations:** Herz- und Diabeteszentrum NRW, Institut für Laboratoriums- und Transfusionsmedizin, Bad Oeynhausen, Germany; U.S. Food and Drug Administration, UNITED STATES

## Abstract

Seroprevalence studies can contribute to a better assessment of the actual incidence of infection. Since long-term data for Germany are lacking, we determined the seroprevalence of immunoglobulin G (IgG) antibodies against severe acute respiratory syndrome coronavirus 2 (SARS-CoV-2) in residual plasma samples of 3,759 German regular blood donors between July 2020 and June 2021. Over almost the entire study period, the incidences determined based on our data were higher than those officially reported by the Robert Koch Institute, the public health institute in Germany. Using our serological testing strategy, we retrospectively detected natural infection in 206/3,759 (5.48%; 95% confidence interval (CI): 4.77–6.25) individuals. The IgG seroprevalence ranked from 5.15% (95% CI: 3.73–6.89) in Lower Saxony to 5.62% (95% CI: 4.57–6.84) in North Rhine Westphalia. The analyses of follow-up samples of 88 seropositive blood donors revealed a comparable fast decay of binding and neutralizing anti-SARS-CoV-2 IgG antibodies. The antibody avidity remained at a low level throughout the whole follow-up period of up to 181 days. Interestingly, female donors seem to express a stronger and longer lasting humoral immunity against the new coronavirus when compared to males. **Conclusion:** Overall, our data emphasizes that seroprevalence measurements can and should be used to understand the true incidence of infection better. Further characterization of follow-up samples from seropositive donors indicated rapid antibody waning with sex-specific differences concerning the strength and persistence of humoral immune response.

## 1. Introduction

The official first Coronavirus disease 2019 (COVID-19) case occurred at the end of December 2019 in Wuhan, China. Sequencing analyses revealed the novel severe acute respiratory syndrome coronavirus 2 (SARS-CoV-2) early as the causative agent for the respiratory disease. Due to rapid transmission, the World Health Organization declared the COVID-19 outbreak a global pandemic on March 11, 2020. To date, a hundred million people worldwide have officially been infected by SARS-CoV-2, resulting in millions of deaths. Recent studies estimate a case fatality rate of about 3%, with various factors increasing the risk of severe disease progression [1, 2]. Risk factors include old age, obesity and various other preexisting conditions and comorbidities [3]. Studies also suggest that women are less likely to develop a severe COVID-19 course compared to infected men [4].

Germany experienced the second and third corona wave roughly appearing between October 20 and February 21 [5] and March 21 and June 21 [6], respectively, during the study period. Increasing infection rates during the second wave were countered by non-pharmaceutical interventions, namely partial (November 2020) and extended (December 2020) lockdown measures [7]. As infection numbers increased substantially again in Germany during March, enhanced non-pharmaceutical interventions were implemented in Germany on April 24, 2021. It is assumed that these strict restrictions reduced infection rates by 50 to 70% [6].

A rapid development of COVID-19 vaccines enabled first vaccinations as early as late 2020, with uneven global supplies [8]. Vulnerable groups in Germany, especially the elderly, and health care workers were prioritized for vaccination in the initial phase as part of a national vaccination campaign [9]. It can be assumed that the number of unreported infections is high because up to 35.1% of SARS-CoV-2 infections are expected to be asymptomatic or paucisymptomatic [10]. Acute SARS-CoV-2 infections are usually verified by polymerase chain reaction (PCR). However, serological testing is suitable to confirm previous SARS-CoV-2 infections [11] and, thus, has a tremendous importance concerning the broad-based surveillance of COVID-19. Thereby, the focus should be on the detection of immunoglobulin G (IgG) antibodies, as these seem to remain detectable over a period of up to 15 months post SARS-CoV-2 infection [12]. By contrast, IgM antibodies peak at an earlier stage and decline rapidly [13]. Most commercially available assays are conceived to detect antibodies against the SARS-CoV-2 spike protein. However, an additional determination of antibodies directed against the viral nucleocapsid is suitable to distinguish between vaccinated and convalescent individuals. This is because administered COVID-19 vaccines only lead to the expression of antibodies against the spike, whereas natural infection induces broader immune response to different viral proteins [14].

Our initial published data revealed a low seroprevalence of 0.91% (95% confidence interval [CI]: 0.58–1.24) in German blood donors in the initial phase of the pandemic between March and June 2020 [15]. Data for the further course of the pandemic are lacking but could lead to a more precise overview of the actual incidence of infection in Germany. In this follow-up study, we, therefore, determined anti-SARS-CoV-2 seroprevalence in a cohort of 3,759 German blood donors resident in the federal states of North Rhine-Westphalia, Lower Saxony and Hesse over a one-year period from July 2020 to June 2021. Follow-up samples of some of the seropositive individuals (88/206) were collected to determine the persistence of anti-SARS-CoV-2 IgG-binding and -neutralizing antibodies, as well as the antibody avidity for up to 181 days.

## 2. Materials and methods

A total of 16,217 residual plasma samples from 3,759 regular blood donors, donated in the period between July 2020 and June 2021, were screened for the presence of anti-SARS-CoV-2 IgG antibodies. Samples were obtained from donors located in the three German federal states of North Rhine-Westphalia (n  =  1,672), Lower Saxony (n  =  816) and Hesse (n  =  1,271). We used exclusively waste material from routine laboratory diagnostics, therefore the need for informed consent was waived. Anonymized samples were collected in accordance with the German Act on Medical Devices for the collection of human residual material. The study was approved by the ethics committee of the medical faculty of the Ruhr University Bochum (AZ: 2022–884).

### 2.1 Semiquantitative determination of anti-SARS-CoV-2 IgG antibodies

Initial testing was conducted by using the enzyme-linked immunosorbent assay (ELISA) from Euroimmun (Lübeck, Germany) targeting the viral spike protein. Semiquantitative results were calculated as a ratio of the sample extinction over the calibrator extinction. The Euroimmun analyzer I system was used for fully automated measurements, according to the manufacturer’s protocol. Results were interpreted as follows: positive: ratio ≥ 1.1; equivocal: ratio ≥ 0.8 to < 1.1; negative: ratio < 0.8.

### 2.2 Quantitative determination of anti-SARS-CoV-2 IgG antibodies

Initial seropositive samples were firstly verified by using a fully automated assay from Abbott (Wiesbaden, Germany). The chemiluminescent microparticle immunoassay measures the IgG antibodies against the receptor-binding domain of the viral spike protein. Values ≥ 7.1 BAU/ml were defined as positive.

### 2.3 Semiquantitative determination of viral antibodies directed against the nucleocapsid

Finally, a fully automated serological ELISA directed against the viral nucleocapsid was used (Euroimmun, Lübeck, Germany). This approach allowed for an additional distinction to be made between naturally infected and vaccinated individuals, since the latter do not form antibodies against the nucleocapsid. Results were interpreted as follows: positive: ratio ≥ 1.1; equivocal: ratio ≥ 0.8 to < 1.1; negative: ratio < 0.8.

### 2.4 Determination of anti-SARS-CoV-2-neutralizing antibodies

The NeutraLISA™ SARS-CoV-2 Neutralization Antibody Detection KIT (Euroimmun, Lübeck, Germany) was used to detect neutralizing antibodies. The sVNT assay is designed to mimic the virus-host interaction using a purified receptor-binding domain protein and immobilized cell surface receptor, angiotensin-converting enzyme-2. The assay was performed according to the manufacturer’s instructions. In brief, samples were first diluted 1:5 with an ACE2-buffer. The diluted samples were then transferred onto an ELISA-plate, coated with immobilized SARS-CoV-2-S1-/-RBD protein. After incubation (60 minutes, 37°C), wells were washed three times. After adding enzyme-conjugate (Peroxidase-labeled Streptavidin) to each well, the plate was incubated for 30 minutes at RT and washed again. Finally, a substrate-chromogen solution was added, the plate was incubated for 15 minutes at RT and reaction was stopped by adding a stop-solution. Absorbance was determined at 450 nm and was inversely proportional to the concentration of neutralizing antibodies.

Results are expressed as percentage binding-inhibition, whereas values ≥ 35% are considered positive and values ≥ 20 to < 35% are considered equivocal.

### 2.5 Avidity determination of anti-SARS-CoV-2 IgG antibodies

The avidity of viral antibodies was determined by using a modified version of the semiquantitative anti-SARS-CoV-2 ELISA assay (see 2.1), whereby the only difference relates to the sample preparation. In brief, samples are diluted with either phosphate buffer or a 5.5 M urea solution. Results are expressed as a relative avidity index (RAI), defined as:

$$\frac{Extinction\;of\;the\;sample\;with\;urea\;treatment}{Extinction\;of\;the\;sample\;without\;urea\;treatment}*100$$

According to the manufacturer, results were interpreted as follows: high-avidity: RAI ≥ 60%; equivocal avidity: RAI ≥ 40 to < 60%; low-avidity: RAI < 40%.

## 3. Results

### 3.1 Presence of anti-SARS-CoV-2 antibodies in blood donors

**Fig 1:**
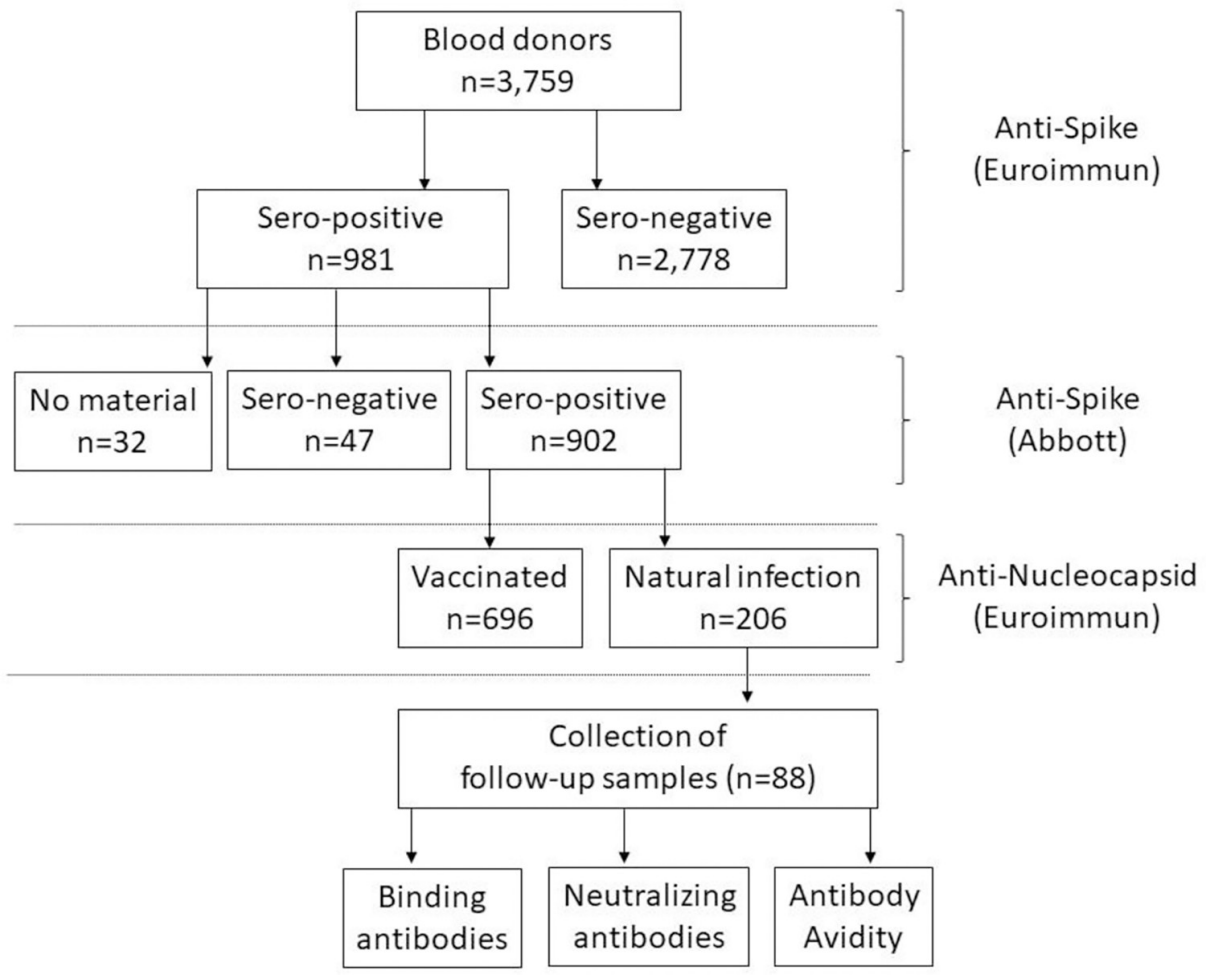
Flowchart illustrating the methodology design of the present study. A total of 3,759 blood donors were tested for anti-SARS-CoV-2 IgG antibodies over a one-year period. Samples were initially tested using a semiquantitative assay. Seropositive results were verified using a quantitative serological test. We additionally performed an assay to detect antibodies against the nucleocapsid, only being expressed by convalescents, to differentiate between vaccinated and convalescent blood donors among the positive samples. Using this approach, we identified 206 naturally infected and 696 vaccinated blood donors within our cohort. We collected and analyzed follow-up samples from 88 naturally infected individuals to assess the strength and persistence of anti-SARS-CoV-2 IgG binding- and neutralizing antibodies as well as the antibody avidity.

**Fig 2:**
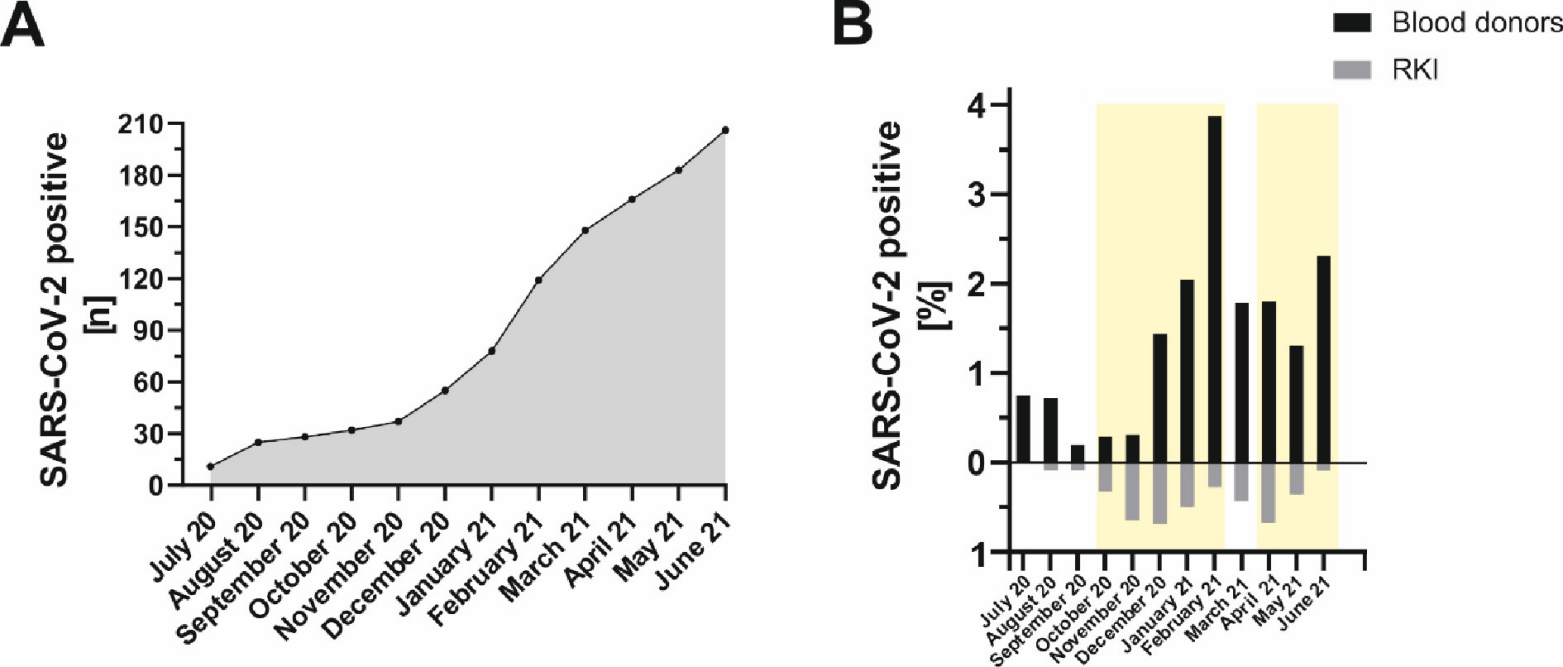
Monthly anti-SARS-CoV-2 antibody detection in the period between July 2020 and June 2021 in blood donors residing in the three German federal states of North Rhine-Westphalia, Lower Saxony and Hesse. A: Cumulative incidence of anti-SARS-CoV-2 IgG seropositive blood donors by month. B: Comparison of percentage anti-SARS-CoV-2 positivity rate among blood donors (seroprevalence, black bars) with officially reported data from the Robert Koch Institute (RKI) [16] (PCR detection, grey bars). The light-yellow areas symbolize the second and third corona wave in Germany.

The primary aim of our study was to determine the anti-SARS-CoV-2 seroprevalence in regular blood donors from three German federal states within a period of one year. Overall, we detected anti-SARS-CoV-2 IgG antibodies formed after natural infection in a total of 206/3,759 blood donors (5.48%; 95% CI: 4.77–6.25) in our cohort throughout the one-year study period (Figs 1 and 2A).

Similarly, the seroprevalence did not differ statistically between the three federal states (p  =  0.628), but incidence was highest in North Rhine-Westphalia (5.62%; 94/1,672; 95% CI: 4.57–6.84), followed by Hesse (5.51%; 70/1,271; 95% CI: 4.32–6.91) and Lower Saxony (5.15%; 42/816; 95% CI: 3.73–6.89). Fig 2B compares the percentage SARS-CoV-2 positivity rate among blood donors (seroprevalence, black bars) with the data officially reported by the Robert Koch Institute, the public health institute in Germany, (PCR detection, light gray bars) for the three German federal states of North Rhine-Westphalia, Lower Saxony and Hesse. As can be seen, the relative incidences determined in our study were far above those that could be calculated based on the officially reported cases in most months between July 2020 and June 2021. The highest seroprevalences, with 3.88% (February 2021) and 2.31% (June 2021), were detected at the end of the second and third corona wave (Fig 2B, light yellow areas) in Germany, respectively. The vaccination rate among blood donors increased steadily during the period between February and June 2021, with 18.52% (696/3,759; 95% CI: 17.29–19.79) of individuals being vaccinated by the end of the study (S1 Fig).

### 3.2 Characterization of seropositive blood donors

In addition to anti-SARS-CoV-2 IgG-binding antibodies, plasma samples of 88 (male: n = 58; female: n = 30) seropositive blood donors were subsequently screened for neutralizing antibodies against the virus and the antibody avidity to assess the binding strength. Follow-up samples were used to evaluate the persistence of these parameters during four different timepoints (t0 = initial seropositivity, t1 = up to 50 days after initial seropositivity, t2 = 50–100 days after initial seropositivity and t3 = 100–200 days after initial seropositivity) over a period of up to 181 days.

#### 3.2.1 Persistence of anti-SARS-CoV-2 IgG-binding antibodies

**Fig 3:**
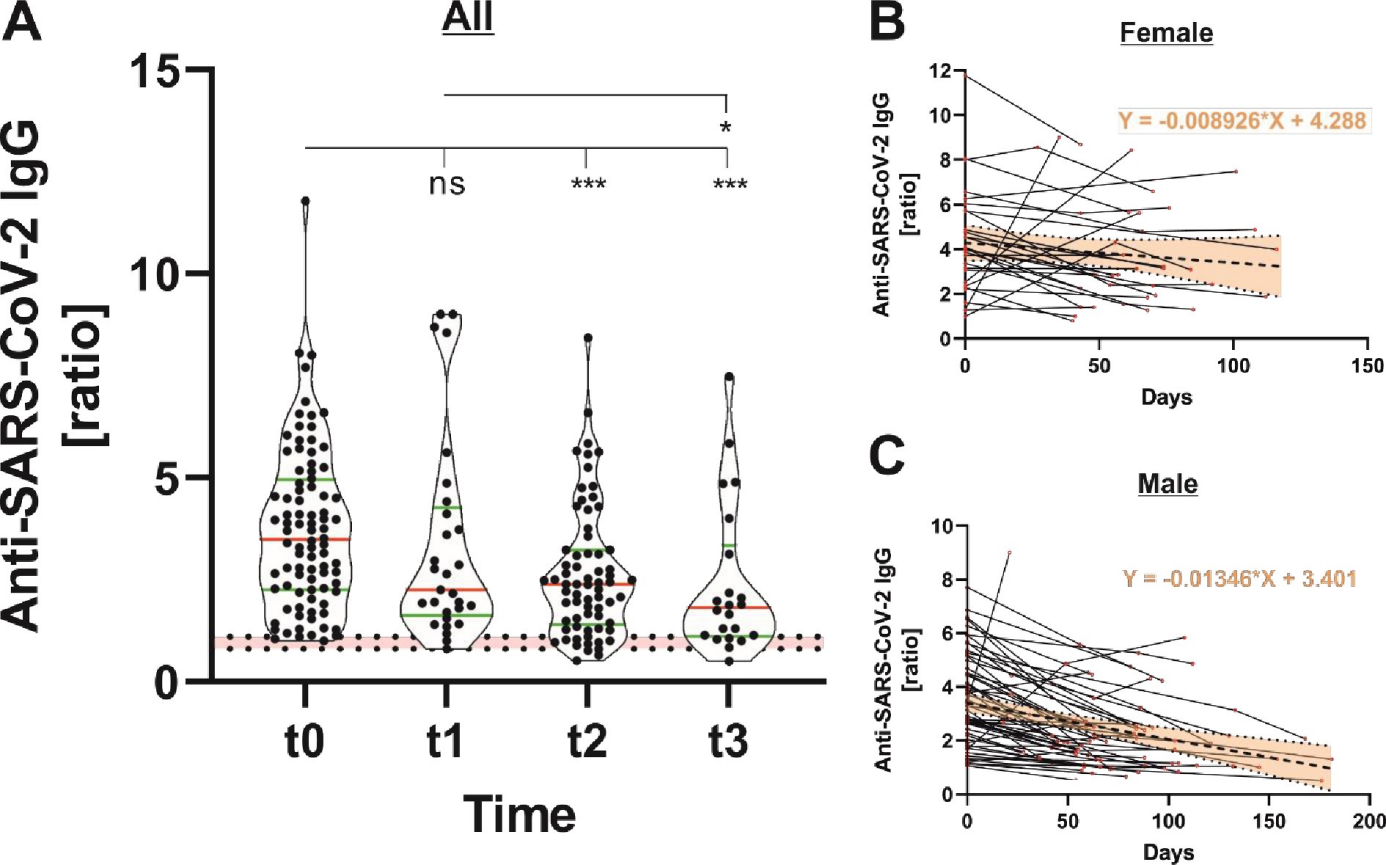
Persistence of anti-SARS-CoV-2 IgG-binding antibodies directed against the viral spike protein (n = 88). Anti-SARS-CoV-2 IgG ratios were determined at different time points (t0 = initial seropositivity, t1 = up to 50 days after initial seropositivity, t2 = 50–100 days after initial seropositivity, and t3 = 100–200 days after initial seropositivity) using a semiquantitative assay from Euroimmun (A). Considering regression line slopes, IgG-binding antibodies were more persistent in female (B) compared to male (C) blood donors.

Only the semiquantitative assay was used for the determination of anti-SARS-CoV-2 IgG-binding antibodies of follow-up samples because of insufficient donor material. Anti-SARS-CoV-2 IgG-binding antibodies declined significantly over time (Fig 3A). Within the first 50 days after initial seropositivity, the median semiquantitative ratio dropped from 3.49 (t1; CI: 2.26–4.95) to 2.26 (t2; CI: 1.64–4.27). The median ratio altered only slightly in the further course from 2.39 (t3; CI: 1.41–3.23) to 1.82 (t4; CI: 1.12–3.35). Considering regression line slopes, anti-SARS-CoV-2 IgG-binding antibodies decreased more strongly in male donors (slope = -0.013) compared to females (slope = -0.0042) (Fig 3B and 3C). The analysis of covariance (ANCOVA) revealed significant differences between the elevations (F = 14.82, DFn = 1, DFd = 205, P = 0.0002).

#### 3.2.2 Persistence of neutralizing antibodies

**Fig 4:**
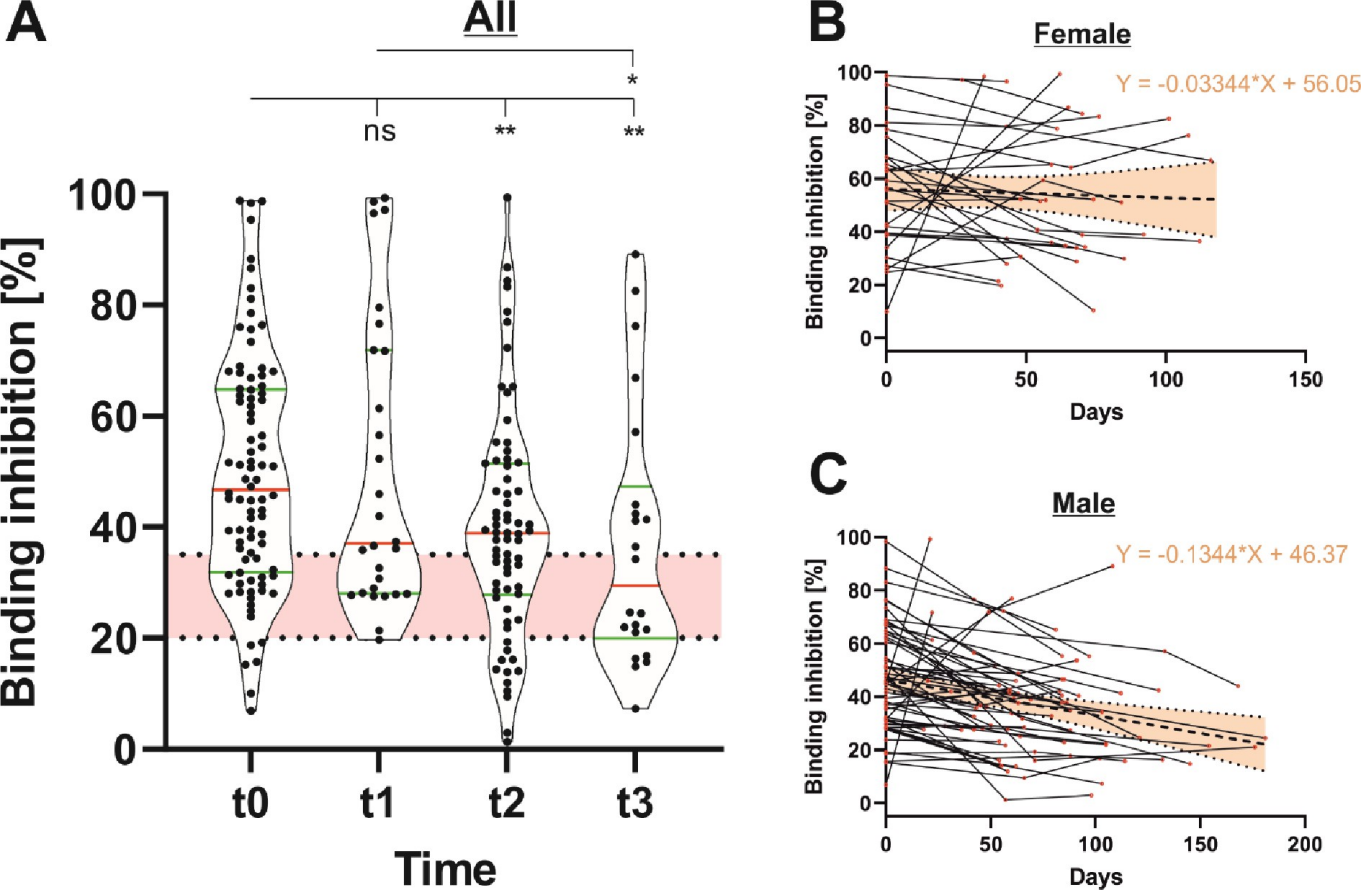
Persistence of anti-SARS-CoV-2-neutralizing antibodies (n = 88). Binding inhibition of anti-SARS-CoV-2-neutralizing antibodies was determined at different time points (t0 = initial seropositivity, t1 = up to 50 days after initial seropositivity, t2 = 50–100 days after initial seropositivity and t3 = 100–200 days after initial seropositivity) using an ELISA-based assay from Euroimmun (A). Considering regression line slopes, neutralizing antibodies were more persistent in female (B) compared to male (C) blood donors.

We additionally determined the neutralizing capability in order to assess the functionality of anti-SARS-CoV-2 IgG antibodies. As shown in Fig 4A, the median binding inhibition capability was at a comparable low level throughout the whole study period. Starting at a median binding inhibition value of 46.70% (CI: 31.89–64.83) at t0, the neutralizing capability of antibodies decreased to 37.03% (CI: 28.00–71.86), 38.84% (CI: 27.78–51.50) and 29.42% (CI: 19.99–47.33) at t1, t2 and t3, respectively. The persistence of neutralizing antibodies was again much stronger in females compared to males, shown by a slope-value of -0.0334 and -0.1344, respectively (Fig 4B and 4C). The ANCOVA analysis revealed highly significant differences between the elevations (F = 19.07, DFn = 1, DFd = 205, P < 0.0001).

#### 3.2.3 Determination of antibody avidity maturation

**Fig 5:**
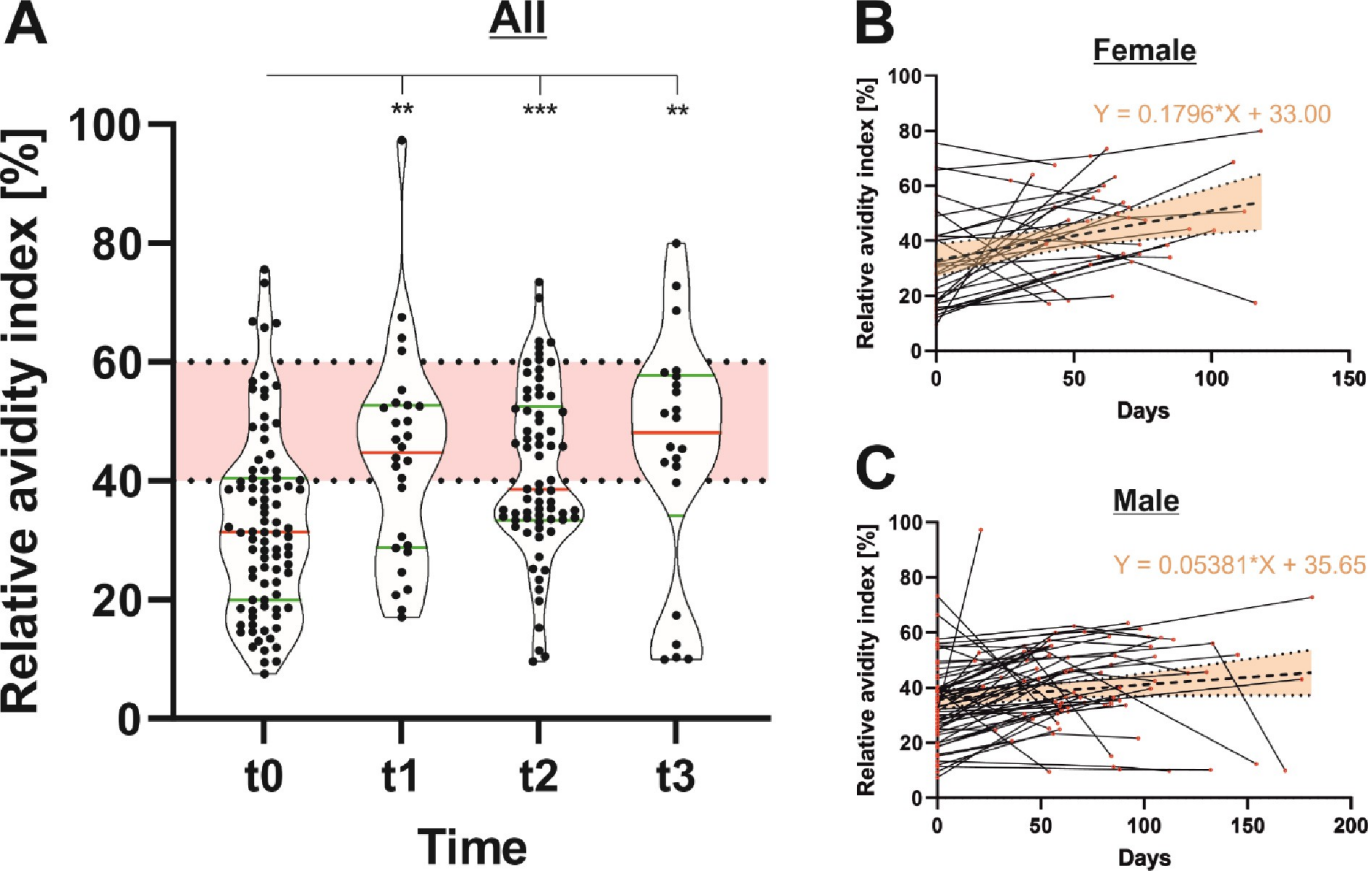
Persistence of anti-SARS-CoV-2 antibody avidity (n = 88). The avidity of anti-SARS-CoV-2 IgG antibodies was determined at different time points (t0 = initial seropositivity, t1 = up to 50 days after initial seropositivity, t2 = 50–100 days after initial seropositivity and t3 = 100–200 days after initial seropositivity) using an assay from Euroimmun (A). Considering regression line slopes, antibody avidity increased more strongly in female (B) compared to male (C) blood donors.

We determined the relative avidity index to appraise the antibody binding strength (Fig 5). While avidity generally increased over time, the levels detected were comparatively low throughout the whole study period. In brief, seropositive tested donors initially showed an average median RAI value of 31.34% (t0, CI: 19.95–40.42), which increased to 44.80% (CI: 28.81–52.67) at t1. After a slight drop at t2 (38.53%, CI: 33.41–52.56), we observed a renewed rise, resulting in a RAI value of 48.15% (CI: 34.13–57.72) at t3. Over time, the avidity has risen more strongly in female donors (slope = 0.1796) compared to male donors (slope = 0.0538). The ANCOVA analysis thereby revealed a significant difference between the slopes (F = 4.245, DFn = 1, DFd = 204, P = 0.0406).

## 4. Discussion

SARS-CoV-2 spread worldwide after the first COVID-19 case occurred in Wuhan, China, in December 2019, leading the World Health Organization to officially declare the infectious disease a pandemic on March 11, 2020. Patients aged > 60 years and those suffering from various medical comorbidities are at increased risk of severe disease progression. By contrast, young and healthy individuals are often asymptomatically or paucisymptomatically infected, resulting in many cases not being detected [10]. In order to acquire a better assessment of the number of undetected infections, we determined the anti-SARS-CoV-2 seroprevalence in a cohort of healthy German blood donors over a one-year period and compared it with the officially reported case numbers of the Robert Koch Institute, the public health institute in Germany. In an earlier study, we detected anti-SARS-CoV-2 antibodies in 0.91% (95% CI: 0.58–1.24) of blood donors screened during the initial phase of the pandemic between March and June 2020 [15]. Seroprevalence data for German blood donors are lacking for the further course of the pandemic, therefore, the aim of the present study was to extend the seroprevalence screening for a prolonged one-year period between July 2020 and June 2021. False-positive measurements could account for a considerable number in populations with a low seroprevalence, thus, initial seropositive measurements were firstly verified by applying one additional assay, both targeting the viral spike protein. No material was available for further analysis out of 32 initial positive samples. Consequently, these samples were not included in the final evaluation. We revised 4.8% (47/981) initially (equivocal) seropositive tested specimens, resulting in 902 samples being seropositive using both assays. COVID-19 vaccines administered in Germany are based on the spike antigen alone, therefore, vaccinated individuals do not express antibodies against the nucleocapsid [17]. We, therefore, subsequently used an assay to detect antibodies directed against the nucleocapsid to distinguish between naturally infected and vaccinated individuals. Using the stepwise approach, we detected antibodies attributable to natural infection in a total of 206/3,759 (5.48%; 95% CI: 4.77–6.25) individuals (Figs 1 and 2) during the observation period from July 2020 to June 2021. Anti-SARS-COV-2 seroprevalence increased steadily from December 2020 within our analyzed blood donor cohort, peaking in February 2021 (positivity rate 3.88%, 95% CI: 2.80–5.22) at the end of the second corona wave in Germany (Fig 2B). The enhanced infection rate is most probably explainable by seasonal effects. Colder temperatures during the winter period lead people to spend more time indoors, which increases viral transmission [18]. The national holidays that occur in Germany at the end of December also facilitate the gathering of larger groups. After seropositivity subsequently decreased within our study group until May 2021, the proof of anti-SARS-CoV-2 antibodies rose again in June 2021 (Fig 2B). The main reason for this observation is probably the appearance of the novel Delta variant of the virus. In Europe, this variant was first detected in the United Kingdom in April 2021 and subsequently became the dominant SARS-CoV-2 strain in most European countries [19]. Compared to the Alpha variant that had predominated until then, the new Delta variant was characterized by increased transmissibility of up to 40–80% [20, 21]. Acute SARS-CoV-2 infections are primarily verified by PCR, whereas seroconversion of anti-SARS-CoV-2 IgG antibodies takes several weeks. Considering this, our data are in line with those officially reported by the Robert Koch Institute based on PCR testing, showing the highest positivity rates between November (0.65%) and December (0.68%) 2020 as well as during April 2021.

Our findings are in line with comparable studies from Germany, indicating low seroprevalences up until November 2020 [22, 23]. In a multilocal study, Gornyk et al. determined seroprevalences in seven German districts between July 2020 and July 2021. Matching our results, authors concluded that seroprevalence rose especially during the second and third COVID-19 waves. Thereby, seroprevalence estimation was 1.3–2.8% between July and December 2020 and rose to 4.1–13.1% between February and May 2021 [23].

Although the differences between the three German federal states considered are not significant, we detected the highest rate of individuals infected with SARS-CoV-2 over the entire study period in North Rhine-Westphalia (5.62%; 94/1,672; 95% CI: 4.57–6.84), followed by Hesse (5.51%; 70/1,271; 95% CI: 4.32–6.91) and Lower Saxony (5.15%; 42/816; 95% CI: 3.73–6.89) (S2 Fig). This could be explained by differences concerning population density, which is much higher in North Rhine-Westphalia (526 inhabitants/km^2^) compared to Hesse (297 inhabitants/km^2^) and Lower Saxony (167 inhabitants/km^2^) [24]. In fact, previous publications have already revealed an association between population density and transmission of the SARS-CoV-2 virus [25, 26].

We further analyzed follow-up samples of 88 seropositive blood donors to evaluate the persistence of anti-SARS-CoV-2-binding and -neutralizing antibodies as well as antibody avidity. It should be noted that blood donors represent a special collective, whereby young and healthy adults, not showing any preexisting conditions, are usually overrepresented. Thus, according to the latest findings, a mild course of the disease can be expected in most cases. The IgG antibodies directed against the viral spike protein decreased steadily during the study period of up to 181 days, whereby the strongest effects were detected within the first 50 days after initial seropositivity (antibody decline of 35%). This is in accordance with previous publications, suggesting a comparable rapid loss of anti-SARS-CoV-2 IgG antibodies after mild infection [27, 28]. In the further course, IgG antibody levels decreased only slightly, whereby the median ratio was consistently above the manufacturer’s cutoff throughout all time points. Yousefi et al. had already showed that anti-SARS-CoV-2 IgG levels decrease steadily, but were still detectable for a long period of at least 15 months [12].

Our data additionally show a decline of neutralizing antibodies, but to a lesser extent compared to anti-SARS-CoV-2 IgG-binding antibodies. Recent studies have revealed high correlations between neutralizing antibody levels and disease progression of infected individuals [29, 30]. Thereby, severe COVID-19 courses seem to lead to much higher anti-SARS-CoV-2 neutralizing antibody titers when compared to mild or asymptomatic COVID-19 infections. In addition, data of Yu et al. suggests much stronger neutralizing antibody induction after vaccination compared to natural immunity [31].

The study of Manuylov et al. recently showed that the avidity of anti-SARS-CoV-2 IgG antibodies can be used as a prognostic factor for the severity of COVID-19 reinfection. A low avidity index (≤ 40%) following the initial infection enhances the risk of a severe COVID-19 course in case of SARS-CoV-2re-infection significantly [32].

As may be expected, our data reveal that the avidity of anti-SARS-CoV-2 IgG antibodies increased significantly during the study period. Nevertheless, median RAI values were at a comparably low level during the whole study period. These findings are in line with those of other research groups, showing an incomplete avidity maturation pattern of SARS-CoV-2 IgG antibodies after natural infection [32–34]. In contrast, Struck et al. show that two vaccination steps or the combination of vaccination and natural infection lead to the establishment of high avidity anti-SARS-CoV-2 IgG antibodies [34].

We determined sex-specific differences regarding antibody characteristics. Female donors initially show significantly higher titers, as well as an extended persistence, of anti-SARS-CoV-2 IgG-binding and -neutralizing antibodies. Furthermore, antibody avidity increases significantly more strongly over time in female compared to male donors. Earlier studies have already suggested a stronger induction of anti-SARS-COV-2 IgG antibodies in females [35]. The apparently reduced humoral immune response may be causative of the generally more severe COVID-19 outcomes as well as higher morbidity and mortality in men compared to women [36].

It should be emphasized that the preselection of blood donors as a study cohort is accompanied by limitations regarding the representation of the population: blood donors are between 18 and 65 years old, young healthy adults are usually overrepresented and other groups (e.g. children, HIV/HCV/HBV-infected patients, older people with underlying conditions, institutionalized people) are excluded or underrepresented. Another limitation of our study is the comparably low number of follow-up samples collected.

Our findings suggest a considerable number of unrecorded SARS-CoV-2 infections within the one-year study period between July 2020 and June 2021 in Germany. As has already mentioned by other groups [37], our data reinforces that longitudinal seroprevalence studies are helpful to assess the actual incidence of infection better within the respectively considered region and time span. Our data additionally reveal a fast decay of binding and neutralizing anti-SARS-CoV-2 IgG antibodies within the early phase after seropositivity and a generally low level of antibody avidity throughout the whole study period. It is noteworthy that sex-specific differences in humoral immunity against the virus were observed.

## Supporting information

S1 Fig Cumulative illustration of individuals vaccinated against SARS-CoV-2 by month. (DOCX)

S2 Fig Monthly anti-SARS-CoV-2 antibody detection in the period between July 2020 and June 2021 in blood donors residing in the three German federal states North Rhine-Westphalia (black circles), Hesse (dark-grey squares) and Lower Saxony (light-grey triangles). (DOCX)
