# Supplementary figures and images for: Analysis of a German blood donor cohort reveals a high number of undetected SARS-CoV-2 infections and sex-specific differences in humoral immune response
Source: PLoS One. 2022 Dec 16;17(12):e0279195. doi: 10.1371/journal.pone.0279195 (PMC9757571; doi:10.1371/journal.pone.0279195)

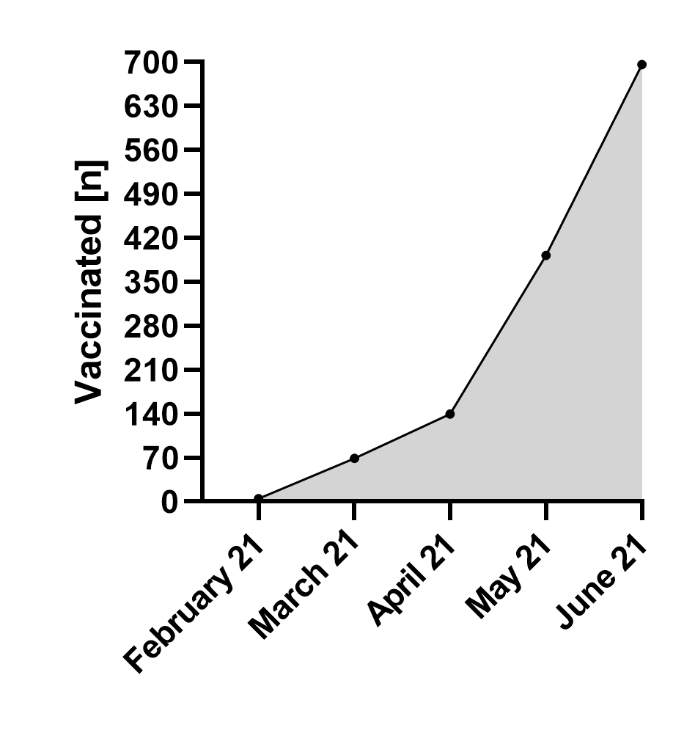


S1 Fig. Cumulative illustration of individuals vaccinated against SARS-CoV-2 by month.

Supplement: S1 Fig — (DOCX) [file pone.0279195.s001.docx]
