# Supplementary material for: Analysis of a German blood donor cohort reveals a high number of undetected SARS-CoV-2 infections and sex-specific differences in humoral immune response
Source: PLoS One. 2022 Dec 16;17(12):e0279195. doi: 10.1371/journal.pone.0279195 (PMC9757571; doi:10.1371/journal.pone.0279195)
Supplement: S2 Fig — (DOCX) [file pone.0279195.s002.docx]

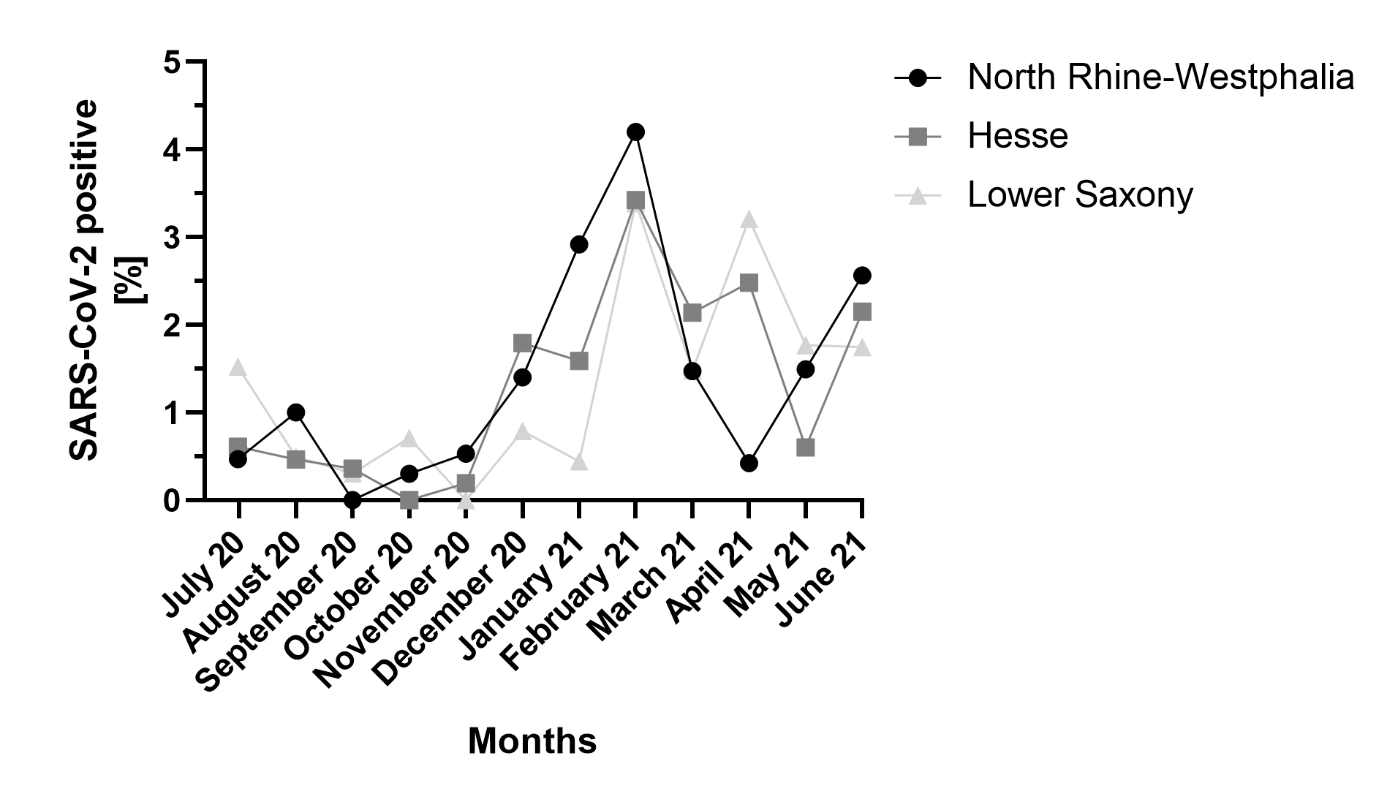


S2 Fig. Monthly anti-SARS-CoV-2 antibody detection in the period between July 2020 and June 2021 in blood donors residing in the three German federal states North Rhine-Westphalia (black circles), Hesse (dark-grey squares) and Lower Saxony (light-grey triangles).
